# Large-scale analyses of heat shock transcription factors and database construction based on whole-genome genes in horticultural and representative plants

**DOI:** 10.1093/hr/uhac035

**Published:** 2022-02-19

**Authors:** Tong Yu, Yun Bai, Zhuo Liu, Zhiyuan Wang, Qihang Yang, Tong Wu, Shuyan Feng, Yu Zhang, Shaoqin Shen, Qiang Li, Liqiang Gu, Xiaoming Song

**Affiliations:** 1 School of Life Sciences, North China University of Science and Technology, Tangshan 063210, Hebei, China; 2 Faculty of Life Science, Tangshan Normal University, Tangshan 063000, Hebei, China; 3 School of Life Science and Technology, University of Electronic Science and Technology of China, Chengdu 610054, China; 4 Food Science and Technology Department, University of Nebraska-Lincoln, Lincoln, NE 68588, USA

## Abstract

Heat shock transcription factor (*Hsf*) plays a critical role in regulating heat resistance. Here, 2950 *Hsf* family genes were identified from 111 horticultural and representative plants. More *Hsf* genes were detected in higher plants than in lower plants. Based on all *Hsf* genes, we constructed a phylogenetic tree, which indicated that *Hsf* genes of each branch evolved independently after species differentiation. Furthermore, we uncovered the evolutionary trajectories of *Hsf* genes by motif analysis. There were only six motifs (M1–M6) in lower plants, and then four novel motifs (M7–M10) appeared in higher plants. However, the motifs of some *Hsf* genes were lost in higher plants, indicating that *Hsf* genes have undergone sequence variation during their evolution. The number of *Hsf* genes lost was greater than the number of genes that were duplicated after whole-genome duplication in higher plants. The heat response network was constructed using 24 *Hsf* genes and 2421 downstream and 222 upstream genes of *Arabidopsis*. Further enrichment analysis revealed that *Hsf* genes and other transcription factors interacted with each other in the response to heat stress. Global expression maps were illustrated for *Hsf* genes under various abiotic and biotic stresses and several developmental stages in *Arabidopsis*. Syntenic and phylogenetic analyses were conducted using *Hsf* genes of *Arabidopsis* and the pan-genome of 18 *Brassica rapa* accessions. We also performed expression pattern analysis of *Hsf* and six *Hsp* family genes using expression values from different tissues and heat treatments in *B. rapa*. The interaction network between the *Hsf* and *Hsp* gene families was constructed in *B. rapa*, and several core genes were detected in the network. Finally, we constructed an *Hsf* database (http://hsfdb.bio2db.com) for researchers to retrieve *Hsf* gene family information. Therefore, our study will provide rich resources for the study of the evolution and function of *Hsf* genes.

## Introduction

Nowadays, nearly all plants often suffer from several abiotic stresses under a vast range of different environments [1, 2]. Generally, the abiotic stresses mainly include drought, high salinity, water deficiency, radiation, acidity, and low or high temperature [1–3]. Among these extreme stresses, high temperature is one of the most important factors as it affects plant distribution [4]. Heat stress also affects plant photosynthesis, induces cellular death, and destroys cell membranes [5, 6]. In response to heat stress, plants need to alleviate its adverse effects to maintain their yield or production quality [7, 8]. Therefore, plant growth and development need to adapt to heat stress [9, 10]. It is of critical importance to control the expression of stress-responsive genes to adapt to heat stress [11–13]. *Hsf* is a key transcription factor gene family, which responds to heat stress and plays an important role in heat resistance [14–16].

Previous reports showed that *Hsf* family genes contained several conserved domains [17]. At the N-terminus, there was a DNA-binding domain (DBD), which could recognize the elements of promoters in heat-response genes. An adjacent oligomerization domain (OD or HR-A/B) was found in all *Hsf* family genes, and this domain mainly comprised hydrophobic heptad repeats [18, 19].

An *Hsf* family gene was first cloned and characterized in *Saccharomyces cerevisiae* (yeast) [20]. Until now, the *Hsf* gene family has been detected in the whole genome in most species, such as in *Arabidopsis thaliana* (21 genes) [9], *Oryza sativa* (25) [9], *Solanum lycopersicum* (26) [21], *Brassica oleracea* (35) [22], *Brassica rapa* (36) [17, 23], *Brassica juncea* (60) [24], *Brassica napus* (64) [25], *Apium graveolens* (17) [26], *Coriandrum sativum* (32) [26], *Daucus carota* (14) [26], *Lactuca sativa* (32) [26], *Capsicum annuum* (25) [27], *Cicer arietinum* (20) [28], *Manihot esculenta* (32) [29], *Vitis vinifera* (19) [30], *Prunus mume* (18) [31], *Sesamum indicum* (30) [32], *Glycine max* (38) [33], *Fagopyrum tataricum* (29) [34], *Zea mays* (31) [35], and *Triticum aestivum* (61) [36]. All of these studies provided rich resources for comparative analysis of the *Hsf* gene family in plants.

Currently, with the release of more and more species genomes, it is possible to identify a large number of *Hsf* family genes at the whole-genome level in different species. Several databases have been constructed to collect the transcription factors, such as the plant transcription factor database (PlantTFDB, http://planttfdb.gao-lab.org/) [37]. However, this database was not specifically made for the *Hsf* gene family, and much detailed information and comparative analysis is absent. In addition, a heat shock protein information resource (HSPIR, http://pdslab.biochem.iisc.ernet.in/hspir/) was constructed to collect information on heat shock proteins [38]. This database contains six major heat shock proteins, but does not contain the *Hsf* gene family. Most existing databases have not been updated with the newest information on genome sequences. Compared with existing databases, all the selected species used in our database have complete genome sequencing.

More importantly, we not only provide more comprehensive *Hsf* gene family information through constructing a database but have also carried out large-scale systematic and comprehensive comparative analyses of the *Hsf* gene family in 111 plants to explore their expression pattern and evolutionary mechanism.

## Results

### Identification of the *Hsf* gene family in 111 plants

**Figure 1:**
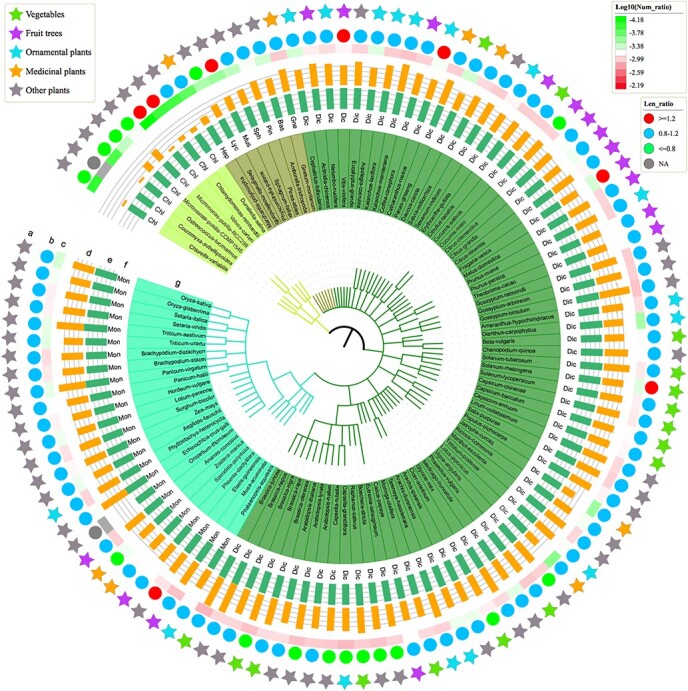
Comparative analysis of *Hsf* family genes in 111 plants. (a) Classification of 111 plants, including horticultural plants (vegetables, fruit trees, ornamental plants, and medicinal plants) and other representative plants. (b) Length ratio of *Hsf* family genes compared with whole-genome genes in each species. (c) Log_10_ number ratio of *Hsf* family genes compared with whole-genome genes in each species. (d) Log_2_ number of *Hsf* family genes in each species. (e) Log_10_ number of whole-genome genes in each species. (f) Classification of each species. (g) Latin name of each species.

Here, we identified a total of 2950 *Hsf* family genes from 3 911 383 gene sequences of 111 species (Fig. 1; Table S1). All of these species have complete whole-genome sequencing, and could well represent the main taxa of plants. The examined species contained 8 lower plants and 103 higher plants. The higher plants were further divided into 71 eudicots, 25 monocots, and 7 other higher plants (1 basal angiosperms, 2 Gymnospermae, 1 Lycopodiophyta, 2 Bryophyta, and 1 Marchantiophyta).

Among these plants, more than a half of the species are horticultural plants (Fig. 1; Table S1), including 16 vegetables (*B. rapa*, *B. juncea*, *B. oleracea*, *Capsicum baccatum*, *C. annuum*, *Capsicum chinense*, *D. carota*, *Cucumis sativus*, *Solanum tuberosum*, *Solanum melongena*, *S. lycopersicum*, *C. arietinum, Beta vulgaris, Moringa oleifera, Phaseolus vulgaris*, and *Raphanus sativus*), 16 fruit trees (*Citrus clementina*, *Citrullus lanatus*, *Citrus grandis*, *Citrus sinensis*, *Cucumis melo*, *V. vinifera*, *Fragaria vesca*, *Malus domestica*, *Prunus persica*, *Musa acuminata*, *Actinidia chinensis*, *Coffea canephora*, *Theobroma cacao*, *Ananas comosus*, *Phoenix dactylifera*, and *Carica papaya*), 16 ornamental plants (*P. mume*, *Catharanthus roseus*, *Amaranthus hypochondriacus*, *Arachis duranensis*, *Capsella grandiflora*, *Cephalotus follicularis*, *Dianthus caryophyllus*, *Kalanchoe laxiflora*, *Kalanchoe marnieriana*, *Eerythranthe guttata*, *Nelumbo nucifera*, *Tarenaya hassleriana*, *Trifolium pratense*, *Phalaenopsis equestris*, *Phyllostachys heterocycla*, and *Aquilegia coerulea*), and 8 medicinal plants (*Salvia miltiorrhiza*, *Panax ginseng, Lotus japonicas, Spirodela polyrhiza, Zostera marina, Gnetum montanum, Jatropha curcas*, and *Marchantia polymorpha*).

The average *Hsf* family gene number was 26.58, and most species (97, 87.39%) had >10 *Hsf* genes (Fig. S1a). We further compared the average length of *Hsf* family genes and all genes of the whole genome in each species (Fig. S1b). The length of *Hsf* family genes was 1.2 times greater than the length of all genes in only 7.21% of plants.

### Comparative analysis of the *Hsf* gene family in plants

**Figure 2:**
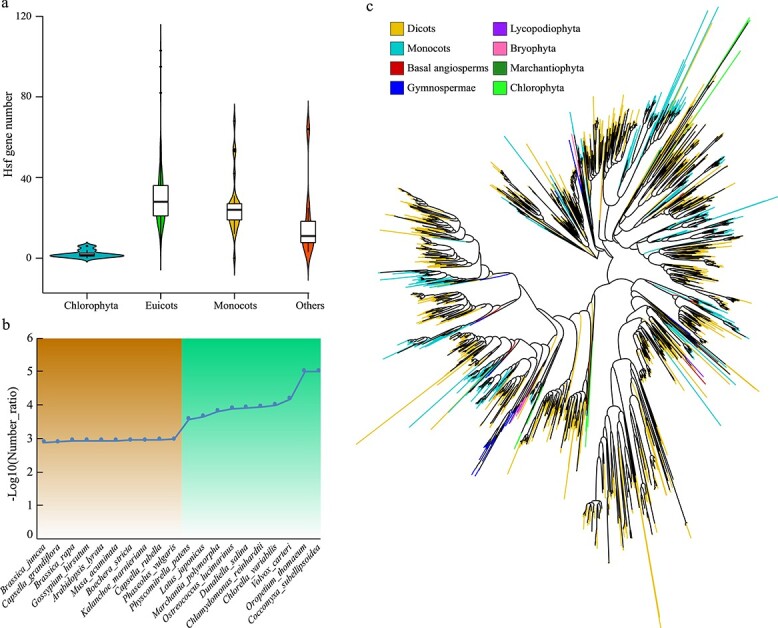
Comparative and phylogenetic analysis of *Hsf* family genes in plants. **a** Boxplot of *Hsf* gene number for different categories of plants. **b** The −log_10_ number ratio of *Hsf* family genes compared with whole-genome genes in representative plants. Orange color indicates the 10 plants with the highest number ratio of *Hsf* family genes, and green indicates the 10 plants with the lowest number ratio of Hsf family genes. **c** Phylogenetic tree using the protein sequences of all the *Hsf* family genes (2950) from 111 plants.

More *Hsf* family genes were detected in higher plants than in lower plants (Figs 1 and 2a). Among the top 10 species with a higher percentage of *Hsf* family genes, all species belonged to the higher plants, including 9 eudicots and 1 monocot (Fig. 2b; Table S1). The only monocot species was *M. acuminata* (banana), which is a tropical fruit and might need more heat-resistant genes to adapt to a high-temperature environment. Interestingly, all three species with the highest percentage of *Hsf* family genes belonged to the Brassicaceae, including *B. juncea*, *C. grandiflora*, and *B. rapa* (Fig. 2b). This phenomenon suggested that the Brassicaceae family might contain a higher proportion of heat-resistant genes than other families.

Among the top 10 species with a lower percentage of *Hsf* family genes, most species (6) belonged to the lower plants (Fig. 2b; Table S1). No *Hsf* family genes were detected in two species, *Coccomyxa subellipsoidea* (lower plant) and *Oropetium thomaeum* (monocot). Besides, the three species with the lowest percentage of *Hsf* family genes were all lower plants: *Volvox carteri*, *Chlorella variabilis*, and *Chlamydomonas reinhardtii*. In *V*. *carteri*, only 1 *Hsf* family gene was detected from the 15 285 genes of the whole genome, only accounting for 0.0065% of all genes. This phenomenon indicated that these species might be using ways to regulate heat stress other than using *Hsf* family genes.

To explore the evolution of the *Hsf* gene family in plants, we constructed a phylogenetic tree using all 2950 protein sequences of the *Hsf* gene family from 111 plants (Fig. 2c). The results showed that most branches contained *Hsf* genes from various plant taxa, and *Hsf* genes of the lower-level species were located at the base of the branches in the phylogenetic tree (Fig. 2c). This phenomenon indicated that the *Hsf* genes of each branch evolved independently after species differentiation. However, there were also some branches that only contained the *Hsf* family genes of monocots and dicots, and they could be clearly separated (Fig. 2c). These results indicated that the *Hsf* genes might have expanded or produced sequence variation in angiosperms.

**Figure 3:**
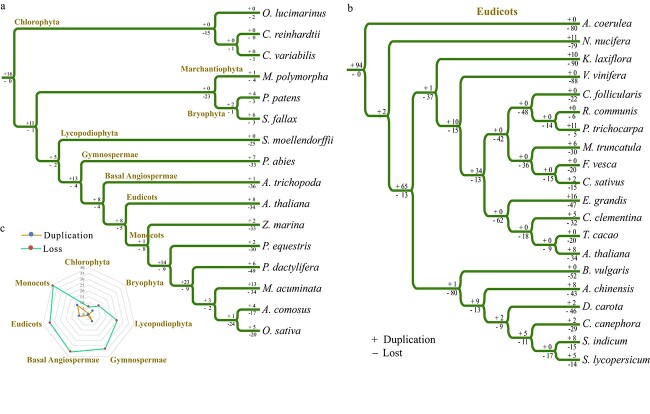
Gene duplication and loss analysis of *Hsf* family genes. **a** Gene duplication and loss analysis of the *Hsf* gene family using Notung software in representative plants. Gene duplications and losses are indicated by numbers with + and −, respectively, on each branch. **b** Gene duplication and loss analysis of the *Hsf* gene family in represent eudicots. **c** Summary of gene duplication and loss numbers in representative plants.

### Gene duplication and loss inference in the *Hsf* gene family in plants

Gene family duplication or loss has often occurred in plants, led by whole-genome duplication (WGD) or whole-genome triplication (WGT) events [39–41]. To clarify the evolution of the *Hsf* gene family, we performed gene duplication and loss analysis in 16 representative plants, including three Chlorophyta, one Marchantiophyta, two Bryophyta, one Lycopodiophyta, one Gymnospermae, one basal Angiospermae, one eudicot, and six monocots (Fig. 3a). The duplication and loss analysis was conducted using Notung software through reconciliation between gene and species phylogenetic trees. Here, we obtained the number of variations of *Hsf* family genes at different stages of evolution according to the reconstructed phylogenies.

In the lineage leading to the common ancestor of all 16 plants, 16 ancestral genes were duplicated, while no gene was lost (Fig. 3a). However, there were 15 gene losses, while no gene was duplicated in the lineage of the common ancestor of three Chlorophyta. Similarly, there were 23 gene losses, while no gene was duplicated in the lineage of the common ancestor of three Bryophyta. In the lineage of the common ancestor of bryophytes and other higher plants, there were 11 gene duplications but only one gene loss. Although most WGD and WGT events occurred in most higher plants, losses of *Hsf* family genes were more than duplications in all the representative Lycopodiophyta, Gymnospermae, and Angiospermae species (Fig. 3a and c). This phenomenon indicated that the losses of *Hsf* family genes were accompanied by WGD or WGT events during plant evolution.

Furthermore, we also conducted *Hsf* family gene duplication and loss analysis in 20 representative eudicot species (Fig. 3b). In the lineage leading to the common ancestor of all these 20 plants, 94 genes were duplicated, while no gene was lost. However, the number of losses of *Hsf* genes was more than gene duplication in most representative eudicots species except *Populus trichocarpa*. This phenomenon further indicated that *Hsf* family genes were lost after WGD or WGT events in most eudicot plants.

### Phylogenetic, conserved motif, and evolutionary trajectory analyses of *Hsf* family genes in seven representative species

**Figure 4:**
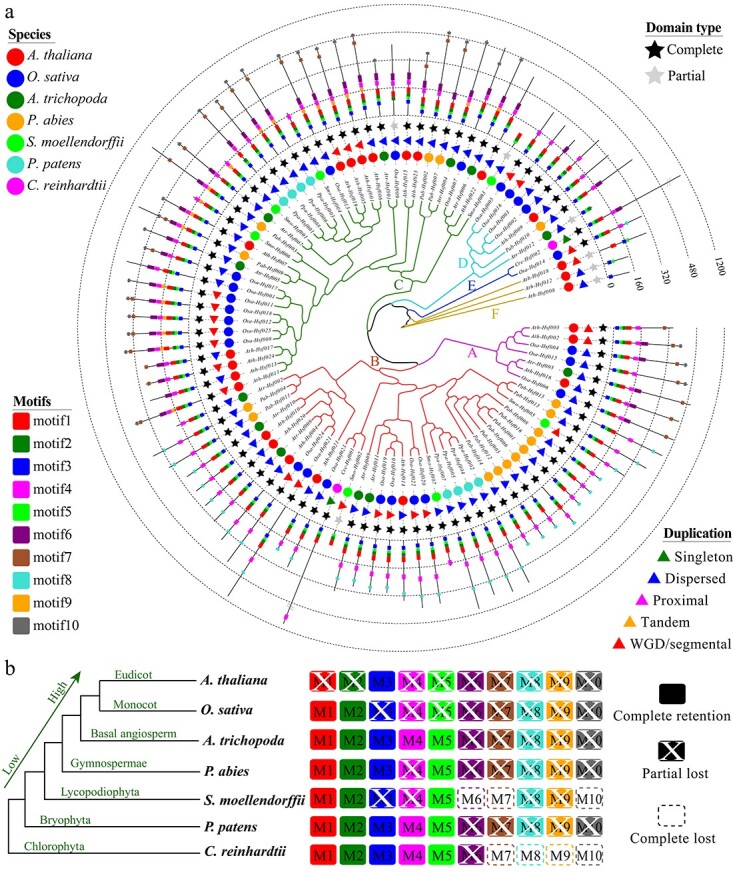
Phylogenetic, conserved motif, duplication type, and evolutionary trajectory analyses of *Hsf* family genes from seven representative species. **a** Maximum-likelihood trees were generated based on the amino acid sequences of the *Hsf* gene family. The tree was constructed using FastTree software. The motifs were identified using the MEME program. The duplication type was detected by the MCScanX program. **b** Evolutionary trajectories of the *Hsf* gene family. The white X indicates that the motif was lost in some genes. The dashed box indicates that the motif was completely lost or did not exist.

Conserved motif analysis can uncover the conservative patterns of a gene family. Here, we explore the conserved motif of *Hsf* family genes in seven representative plants, ranging from a lower plant (*C. reinhardtii*) to higher plants, including the eudicot model species *A. thaliana*, the monocot model species *O. sativa*, the basal angiosperm species *Amborella trichopoda*, the Gymnospermae species *Picea abies*, the Lycopodiophyta species *Selaginella moellendorffii*, and the Bryophyta species *Physcomitrella patens* (Fig. 4).

In total, 94 *Hsf* family genes were identified from the whole genomes of seven species. The largest number of genes was detected in *O. sativa* (25), followed by *A. thaliana* (24) and *P. abies* (16) (Fig. 4a; Fig. S2a). However, there were only two *Hsf* family genes in the lower plant *C. reinhardtii*. To explore the phylogenetic relationship and classification of *Hsf* family genes, we constructed a tree using amino acid sequences. Our analysis revealed that all the *Hsf* family genes could be divided into seven groups, and we defined them as groups A–F (Fig. 4a). There were 36 and 39 *Hsf* family genes in groups B and C, respectively, i.e. more than in the other four groups (Fig. S2b).

Ten motifs [motifs (M) 1–10] were detected in the *Hsf* family genes using the MEME program (Fig. 4a; Table S2). M6 was the longest, followed by M1 and M4 (Fig. S2c, Fig. S3). Four motifs, M3, M5, M2, and M1, were present in almost all *Hsf* family genes. However, some of these motifs were absent in genes *Ath*-*Hsf008*, *Ath*-*Hsf012*, and *Ath*-*Hsf018*. Furthermore, the Pfam analysis showed that the domain of these three genes was also partial in *Arabidopsis* (Fig. 4). Similar, another four genes also had the partial domain, including *Atr-Hsf012*, *Smo-Hsf002*, *Smo-Hsf003*, and *Osa-Hsf009* (Fig. S2d). In addition, we also found some motifs specific to the related groups. For example, 7M8 was detected in almost all *Hsf* family genes of group B, while it was nearly absent in genes of other groups. Similarly, M7 and M10 were only detected in the *Hsf* genes of groups A and C. These motifs might be associated with the functional specificity of different groups of *Hsf* family genes. In conclusion, these results indicate that the motifs in the same group were highly similar, which is consistent with the phylogenetic relationship of these genes.

Furthermore, we tried to explore the evolutionary trajectories of the *Hsf* family gene domains (Fig. 4b). In the lower plant *C. reinhardtii*, only six motifs (M1–M6) were detected, while four motifs (M7–M10) were complete lost. In the bryophyte *P. patens*, all 10 motifs were present, but M6–M10 were partially lost in some *Hsf* genes. Then, we found that M6, M7, and M10 were completely lost in the Lycopodiophyta plant *S. moellendorffii*. In Gymnospermae and angiosperms, all the motifs were present, while some motifs were partially lost in some genes. In *A. thaliana*, only M3 was completely retained in all *Hsf* genes. Similarly, only M1 and M2 were completely retained in all *Hsf* genes in *O. sativa.* This phenomenon indicated that *Hsf* family genes generated sequence divergence during the evolution of the species.

### Duplication type of *Hsf* gene family in seven representative species

**Figure 5:**
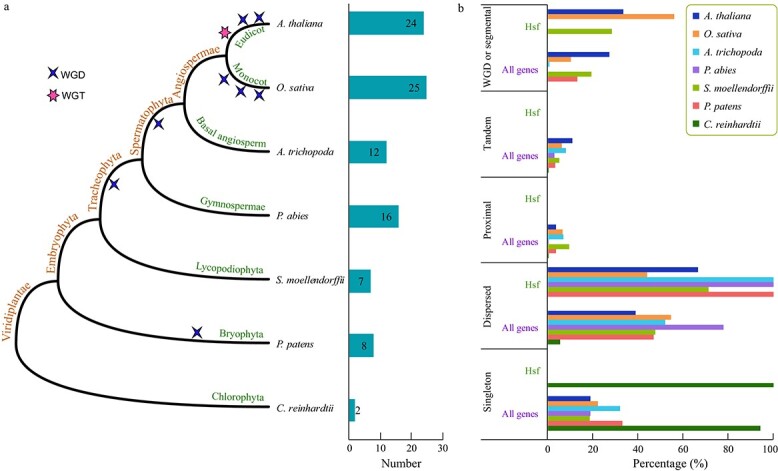
The number of *Hsf* family genes and duplication type for each representative species. **a** The phylogenetic tree and the number of *Hsf* family genes in seven species. **b** The number of each duplication type for *Hsf* family genes and all whole-genome genes in seven species.

We explored the gene family expansion mechanism through gene duplication type analysis. Five types of gene duplication were identified for whole-genome genes and *Hsf* family genes, including singleton, dispersed, proximal, tandem, and WGD/segmental (Figs 4 and 5; Table S3). No *Hsf* family gene belonged to the singleton, proximal, and tandem duplication type in most species except *C. reinhardtii*. The two *Hsf* genes of *C. reinhardtii* were of the singleton type, which was different from the other six higher plants. However, all the *Hsf* genes from *A. trichopoda*, *P. abies*, and *P. patens* were of the dispersed type.

Several *Hsf* genes of *A. thaliana*, *O. sativa*, and *S. moellendorffii* belonged to the WGD/segmental duplication type (Figs 4 and 5; Table S3). The percentage of *Hsf* genes of the WGD/segmental type was 56.00 in *O. sativa* (Table S4). This ratio was significantly higher than the average for whole-genome genes (10.27%) belonging to the WGD/segmental type (*P* < .01). Therefore, the WGD/segmental type played critical roles in the *Hsf* gene family expansion of *O. sativa*. However, we did not find other duplication types for *Hsf* gene family expansion in other species.

### Interaction network construction using target genes of the *Hsf* gene family

**Figure 6:**
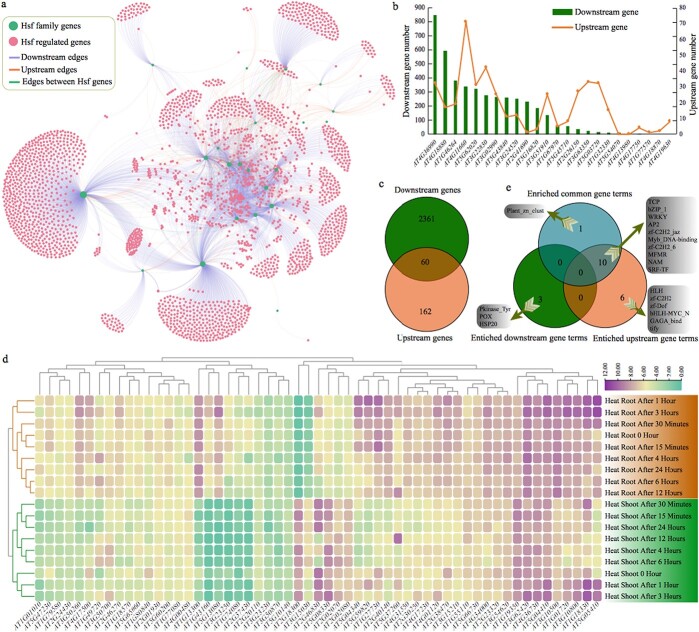
The interaction network among *Hsf* family genes, and their upstream and downstream-regulated genes in *Arabidopsis*. **a** Construction of the network using Gephi software. **b** Number of upstream and downstream genes for each *Hsf* family gene in the network. **c** Specific and shared genes between downstream and upstream genes in the network. **d** Absolute expression values of common target genes up- and downstream from *Hsf* under heat treatment for different lengths of time. **e** Specific and shared terms among upstream, downstream, and common gene enriched terms.

Here, we constructed an interaction network for *Hsf* family genes and their target genes in *Arabidopsis* (Fig. 6a; Table S5). A total of 4788 gene pairs made up the regulatory network, which contained 2421 downstream genes (regulated by *Hsf* family genes) and 222 upstream genes (regulated *Hsf* family genes). We found that the number of target genes regulated by different *Hsf* genes varied greatly. Gene *AT4G36900* had the largest number of downstream genes (845), followed by *AT4G18880* (593) and *AT1G46264* (379) (Fig. 6b; Tables S5–S7). However, there was no downstream gene for three *Hsf* family genes, including *AT1G77570*, *AT4G18870*, and *AT4G19630*. Similarly, gene *AT4G11660* had the largest number of upstream genes (71), while no upstream gene was found for *AT4G13980* and *AT5G54070*.

Interestingly, we found that 60 genes belonged to the downstream and upstream genes, indicating that they were likely to be feedback-regulated genes (Fig. 6c; Table S6). Furthermore, we explored the gene expression patterns of these target genes under heat treatment for different lengths of time (Table S8). The cluster analysis results showed that these genes had obvious tissue-specific expression patterns after heat treatment, i.e. they were clearly divided into two groups according to root and shoot (Fig. 6d). For example, in each heat treatment, the expression level of the *AT1G13300* gene in root was higher than that in shoot. The expression pattern of the *AT2G18300* gene was exactly the opposite of that of *AT1G13300*. The expression level of most genes changed obviously after heat treatment. For example, the expression level of the *AT5G05410* gene increased significantly in root and shoot after treatment for 1 and 3 hours, and then decreased with the extension of the treatment time. This expression map will provide a good reference for further research on the functions of these target genes in heat resistance.

### Functional enrichment analysis of the target genes in the network

To explore the function of the target genes involved the network in *Arabidopsis* constructed above, we conducted enrichment analysis of all the target genes of the *Hsf* gene family. We identified 16 significantly enriched terms (*q*-value <0.05 and fold-change >2) for upstream genes (Table S9). The fold change indicated that the percentage of terms enriched for annotated target genes was comparable to that for all annotated genes. The most significantly enriched term was Apetala 2 (AP2) (*q*-value = 2.30 × 10^−41^ and fold change = 27.29), followed by Myb_DNA-binding and the *TCP* gene family (Table S9). However, only three terms were significantly enriched for the downstream genes. As expected, we found that HSP20 was the most significantly enriched term (*q*-value = 9.82 × 10^−4^ and fold change = 5.22). This phenomenon indicated that the *Hsf* gene family might play more important roles in regulating HSP20 than other heat-shock proteins in *Arabidopsis*. Furthermore, we also conducted enrichment analysis of the 60 common genes, which were assigned to the downstream and upstream genes at the same time. A total of 11 significantly enriched terms were detected, and the most significantly enriched term was No apical meristem (NAM) (*q*-value = 7.48 × 10^−7^ and fold change = 22.63) (Table S9).

Furthermore, we analyzed specific and shared enriched functional terms among 16 upstream, 3 downstream, and 11 common gene terms. As shown by the Venn diagram, six, three, and one enriched functional terms were specific to upstream, downstream, and common genes, respectively (Fig. 6e). By contrast, no significantly enriched terms were detected among these three gene datasets. The results also showed that 10 terms were significantly enriched in upstream and common genes, accounting for 50.0% of all significantly enriched functional terms. Most of these enriched functional terms were related to various stresses, such as AP2, WRKY, zf_C2H2, bZIP, and Myb_DNA-binding. This phenomenon indicated that *Hsf* gene family and other transcription factors interact with each other in response to heat resistance and other stresses in plants.

### Exploring the expression pattern of the *Hsf* gene family under various conditions

Here, we performed expression pattern analysis of the *Hsf* gene family using large-scale expression datasets under various stresses and developmental stages.

**Figure 7:**
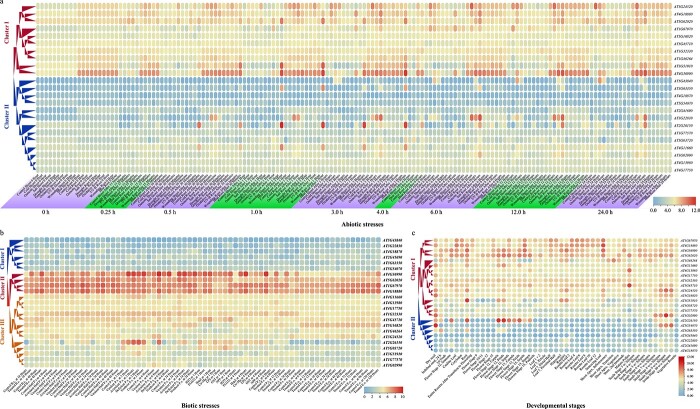
Expression level of *Hsf* family genes obtained from the *Arabidopsis* eFP Browser. **a** Absolute expression values of *Hsf* family genes under various abiotic stresses. **b** Absolute expression values of *Hsf* family genes under various biotic stresses. **c** Absolute expression values of *Hsf* family genes during various developmental stages in different tissues.

We collected 154 samples from 18 groups under various abiotic stresses, including cold, osmotic, salt, drought, genotoxic, oxidative, UV-B, wounding, and heat stress treatment after 0, 0.25, 0.5, 1.0, 3.0, 4.0, 6.0, 12.0, and 24.0 hours in shoot and root of *Arabidopsis* (Fig. 7a; Table S10). The cluster analysis showed that all the genes could be divided into two groups. On the whole, the genes in cluster I had higher expression values than those in cluster II under most stress treatments. However, we found that gene *AT2G26150* from cluster II had a relatively high expression level under heat treatment after 0.5, 1.0, and 3.0 hours in shoot and root. Similarly, genes *AT2G26150* and *AT3G22830* had a relatively high expression level under salt treatment after 3.0, 6.0, 12.0, and 24.0 hours in the root. In addition, we found that gene *AT4G36990* had higher expression values in root than shoot in most stress treatments.

For biotic stresses, we collected 70 samples from 27 groups under various biotic stresses (Fig. 7b; Table S11). Three groups were obtained by cluster analysis according to gene expression in different biotic stresses. All four genes (*AT4G18880*, *AT1G67970*, *AT5G62020*, and *AT4G36990*) in cluster II had the highest gene expression among the three clusters, followed by cluster III and cluster I.

In addition, we examined the gene expression of 47 samples from different developmental stages of several tissues, including seed, leaf, root, flower, and silique (Fig. 7c; Table S12). Similarly, two groups were obtained by the cluster analysis. Most genes in cluster I had a higher expression level than those in cluster II. However, we found that gene *AT2G26150* from cluster II had a relatively high expression level in the carpels, petals, and sepals of flower stages 12 and 15 in *Arabidopsis*. We also found that gene *AT5G54070* from cluster II had a relatively high expression level in the dry seed and siliques of seed stages 8–10. All of these gene expression patterns in the model species *Arabidopsis* will provide a guide for studying the *Hsf* gene family in other species in the future.

### Pan-genome analysis of *Hsf* gene family in *B. rapa*

**Figure 8:**
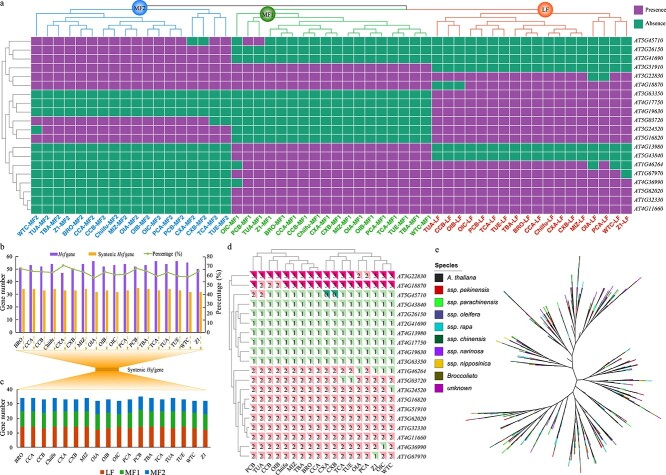
Syntenic and phylogenetic analysis of the *Hsf* gene family in *A. thaliana* and genomes of 18 *B. rapa* accessions. **a** Syntenic genes between *Arabidopsis* and each subgenome (LF, MF1, MF2) of 18 *B. rapa* genomes. **b** Numbers of all *Hsf* family genes and syntenic *Hsf* genes and their ratio in 18 *B. rapa* genomes. **c** Syntenic gene number in each subgenome (LF, MF1, MF2) of 18 *B. rapa* genomes. **d** Heat map of syntenic gene number for each *Arabidopsis Hsf* gene in each of 18 *B. rapa* genomes. **e** Phylogenetic tree using the *Hsf* family genes of *Arabidopsis* and 18 *B. rapa* accessions.

To study *Hsf* gene family variation among different varieties of the same species, we performed *Hsf* gene family analysis in the pan-genome of 18 *B. rapa* accessions (Fig. 8a). A total of 952 *Hsf* family genes were identified in 18 accessions, the number ranging from 47 (CXA accession) to 56 (OIA, TCA, and TUE accessions) in each *B. rapa* accession (Table S13). The syntenic analysis showed that >57.14% of *Hsf* genes were located in the syntenic regions of the genome. The number of *Hsf* family genes in the collinear region varied little among accessions, ranging from 32 to 35, suggesting that the genes in the collinear region were relatively stable (Fig. 8b; Table S13).

Based on gene loss and retention, the *B. rapa* genome was further divided into three subgenomes, the less fractioned subgenome (LF) and the more fractioned subgenomes (MF1 and MF2). The *Hsf* gene number was from 12 to 14 in the LF subgenome, from 9 to 12 in the MF1 subgenome, and from 8 to 9 in the MF2 subgenome (Fig. 8a and c; Tables S13 and S14). Cluster analysis was conducted according to the number of *Hsf* family genes in each subgenome, and the results could be divided into three groups, corresponding to the subgenomes of each genome of 18 accessions (Fig. 8a).

The *B. rapa* genome has experienced an additional WGT event compared with *Arabidopsis*. Among all the *Hsf* family genes, we found that genes *AT3G22830* and *AT4G18870* were well preserved with three copies after duplication in most accessions. However, most *Arabidopsis Hsf* family genes only retained one or two copies in *B. rapa*, which means that some gene loss occurred after the WGT event. Interestingly, we found that the syntenic gene of *AT5G45710* was completely lost in CXA and CXB accessions (Fig. 8d; Tables S14 and S15). The syntenic genes of four *Arabidopsis* genes (*AT1G77570*, *AT3G02990*, *AT4G18880*, and *AT5G54070*) were also completely lost in all 18 *B. rapa* accessions.

Furthermore, we constructed a phylogenetic tree using all the *Hsf* family genes of 18 *B. rapa* accessions and *Arabidopsis* (Fig. 8e). Most branches contained *Hsf* family genes from the *B. rapa* species and *Arabidopsis*. Therefore, we could further explore the evolution and function of homologous genes located on the same branch in *B. rapa* based on *Arabidopsis* genes.

### Phylogenetic and expression analysis of *Hsf* and *Hsp* gene family in *B. rapa*

In addition to identifying 54 *Hsf* family genes in *B. rapa*, we also detected 393 *Hsp* family genes, which were generally regulated by the *Hsf* family genes (Table S16). All of these *Hsp* family genes were further divided into *Hsp20* (38), *Hsp40* (155), *Hsp60* (53), *Hsp70* (34), *Hsp90* (16), and *Hsp100* (97) gene families.

**Figure 9:**
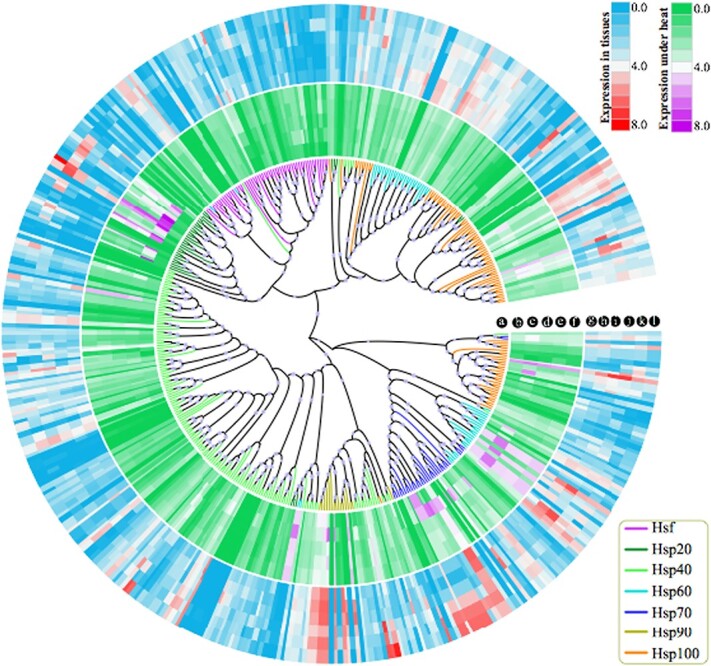
Phylogenetic and expression analyses of the *Hsf* and various *Hsp* gene families in *B. rapa*. (a) Each gene family is marked with a different color in the branches of the phylogenetic tree. The bootstrap was set to 1000 replicates, and values >40% are indicated with a circle. The heat map represents gene expression under different heat treatments (b, control; c, T1; d, T4; e, T8; f, T12) and in different tissues (g, root; h, stem; i, leaf; j, flower; k, silique; l, callus). Expression values are normalized by FPKM, and are log_2_-transformed in the heat map.

To further explore their relationship, we constructed a phylogenetic tree using the protein sequences of these gene families (Fig. 9). The results showed that the *Hsf* gene family had a closer relationship with the *Hsp20* gene family than other families. *Hsp90* had a close relationship with the *Hsp40* gene family, and *Hsp60* had a closer relationship with the *Hsp70* gene family. The *Hsp100* gene family was divided into two branches in the phylogenetic tree, and it had a close relationship with *Hsp60* and *Hsp70*. Therefore, our analysis first clearly revealed their relationship in *B. rapa*, and could be further used to explore their evolution and comparative analysis in *B. rapa* and other plants.

Furthermore, we conducted expression pattern analysis of the *Hsf* and *Hsp* gene families using expression datasets under heat stress in *B. rapa* (Fig. 9; Table S16). For the heat treatment, the samples were treated at 38°C for 1, 4, 8, and 12 hours. The expression heat map shows that several genes from *Hsp20*, *Hsp60*, *Hsp70*, *Hsp90*, and *Hsp40* had highest expression at 1 hour of treatment, and expression then gradually decreased, such as for *Hsp20* family genes *BraA01g018560*, *BraA02g010780*, *BraA03g011180*, and *BraA10g017550* (Fig. 9). We also found that some genes had higher expression under all heat treatments than control, such as *BraA03g027160* (Hsp20) and *BraA06g020450* (Hsp20). The genes in other families showed trends similar to those of the *Hsp20* gene family.

We also performed expression pattern analysis of the *Hsf* and *Hsp* gene families using the expression datasets from different tissues, including root, stem, leaf, flower, silique, and callus of *B. rapa* (Fig. 9; Table S17). By integrating the expression pattern heat map of heat treatment and various tissues, we found that several genes showed high expression in both expression maps, such as genes in the *Hsp60* family (*BraA07g018460* and *BraA08g000690*), *Hsp70* (*BraA03g016490* and *BraA03g019440*), Hsp90 (*BraA01g014950*, *BraA02g013110*, and *BraA06g038950*) and *Hsp100* (*BraA03g015750* and *BraA07g032970*) (Fig. 9).

Overall, this expression pattern map will provide a guide for the study of their function and regulatory network in *B. rapa*.

### Interactive network between *Hsf* and *Hsp* gene families in *B. rapa*

**Figure 10:**
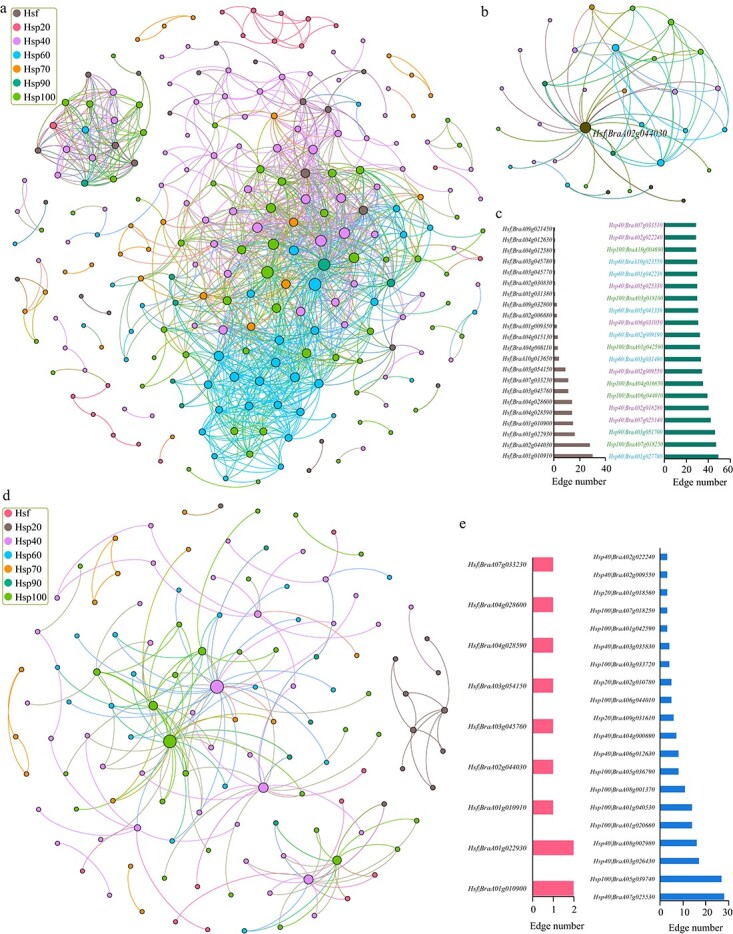
Interaction network analysis of genes of the *Hsf* family and each *Hsp* family in *B. rapa*. **a** Interaction network of *Hsf* and various *Hsp* gene families. Each family is marked with a different color in the circle nodes. All connections (edges) in the network represent PCC values >.95 (positively regulated relationship) or <−.95 (negatively regulated relationship). **b** Interaction network of *Hsf* and *Hsp* gene families with the PCC < −.95. **c** Number of edges formed by *Hsf* and *Hsp* gene families in the network. **d** Interaction network of *Hsf* and various *Hsp* family genes containing HSEs (PCC > .95 or PCC < −.95). **e** number of edges formed by *Hsf* and *Hsp* gene families in the heat response network.

Based on the expression of the *Hsf* and *Hsp* family genes mentioned above, we calculated the Pearson correlation coefficients (PCCs) between any two genes of these gene families, and then constructed regulatory networks. After filtering, a total of 1275 connections (edges) between any two genes in the network with PCC > .95 (Fig. 10a; Table S18). Among these connections, only 65 (5.10%) were negative regulatory with PCC < −.95, while all of the other 1210 connections had a positive relationship (Table S18). This network included 22 *Hsf* and 216 *Hsp* family genes, accounting for 40.74 and 54.96% of all *Hsf* and *Hsp* gene family genes, respectively (Fig. 10a; Table S19). Furthermore, we found that *Hsf* gene *BraA02g044030* had the most negative connections (22) with other *Hsp* family genes, accounting for 33.85% of all 65 negative edges (Fig. 10b; Table S18).

Among *Hsf* family genes, *BraA01g010910* had the most connections (30) with other genes, followed by *BraA02g044030* (28) and *BraA01g022930* (16) (Fig. 10c; Table S19). Among *Hsp* family genes, *BraA01g027780* (Hsp60) had the most connections (49), followed by *BraA07g018250* (*Hsp100*) and *BraA03g051700* (*Hsp90*). Interestingly, most genes (19, 95%) belonged to *Hsp40*, *Hsp60*, and *Hsp100* among the top 20 connections of *Hsp* family genes (Fig. 10c; Table S19). These results indicate that genes with more connections might play a core role in the regulatory network of heat resistance in *B. rapa*.

Furthermore, we predicted the *cis*-acting elements from the promoter sequences of *Hsp* family genes contained in the network. The results showed that the identified *cis* elements were involved in light response, abiotic stress, circadian control, and hormone signaling (Fig. S4, Table S20). This indicated that *Hsp* family genes also participated in a large number of other physiological functions in addition to participating in the regulation of plant heat resistance. This phenomenon is also consistent with previous reports [42–45].

Moreover, we identified the heat shock element (HSE) from the promoter sequences of *Hsp* family genes in the network. Finally, the HSE was detected in 28 *Hsp* family genes, which might play a direct and important role in the regulation of heat resistance. Then, using these genes and the interacting *Hsf* and *Hsp* genes, the heat-resistant regulatory network was reconstructed (Fig. 10d; Table S21). In the network, *BraA07g025530* (*Hsp40*) had the most connections (28) with other genes, followed by *BraA05g039740* (*Hsp100*, 27) and *BraA03g026430* (*Hsp40*, 17) (Fig. 10e; Table S22).

In conclusion, these networks well reveal the regulatory relationship between *Hsf* and various *Hsp* family genes in *B. rapa*, and also lay the foundation for future experimental studies on the functional interaction of these genes.

### Database construction for the *Hsf* family genes

**Figure 11:**
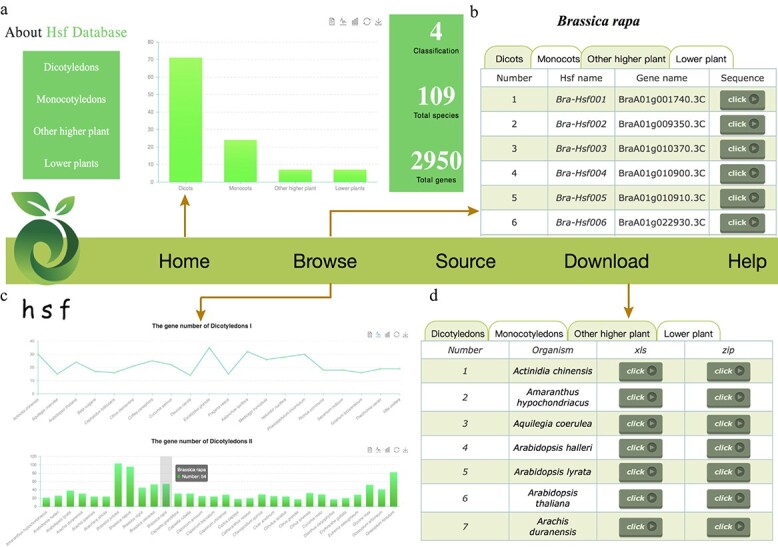
Architecture of the *Hsf* Database, including the home, browse, source, download, and help modules. **a** Home page. **b***Hsf* gene and sequence information of each species in the browse module. **c***Hsf* gene number of each species in the chart function of the browse module. **d** Download function of *Hsf* family genes for each species.

Using these available datasets, we constructed an *Hsf* database, which should help users to easily query, compare, and download *Hsf* family genes of all species simultaneously. All species were taxonomically classified to facilitate checking of the *Hsf* family genes according to their evolutionary relationship. All *Hsf* family gene information was stored in backend tables using MySQL, which could be accessed using the front-end web application of the *Hsf* database. Here, we provide a detailed description of the interfaces of this database, including the home, browse, download, resource, help, and contact interfaces (Fig. 11).

#### Browse

To make it easier for users to check, we further sorted the examined species according to the first letter of their Latin name. The multi-select dropdown allows users to select each species for browsing. We provide detailed information for each species, including the total number, *Hsf* name, gene ID, and protein sequences. Furthermore, we also integrated the search function in the browser interface, which makes it easier for users to find the assigned *Hsf* family gene information according to gene ID.

Besides, the browse home page provides interactive plots and line and bar graphs to view the number of species. Then, bar plots, and line charts are also used to show the *Hsf* family gene number of each species, which makes it easier and faster for users to compare these genes in different species. Finally, all the related information can be downloaded in Excel format. These documents will allow researchers to conduct a local batch comparative analysis of the *Hsf* gene family.

#### Download, resource, and help

The information (Excel format) and sequences (Fasta format) of *Hsf* family genes for each species can be obtained from the download interface. In the resource interface we have collected most *Hsf* research-related databases and provided relevant links for users to easily query and compare studies. In the help interface, we provide users with a detailed *Hsf* database manual. We also provide the e-mail, mobile number, and address to allow users to contact us conveniently.

With the novel genome sequences released in the future, we will continuously identify *Hsf* family genes from these datasets and add them to our *Hsf* database. We also encourage users to submit new *Hsf* family genes to us to further enrich the database. Finally, we welcome feedback from all users for further improvement of our database. We believe that this database will be useful and friendly for all researchers.

## Discussion

In this study, we comprehensively identified 2950 *Hsf* family genes from the whole genome of 111 representative plants, most of which are horticultural plants. Compared with the PlantTFDB, we have also performed *Hsf* gene family analysis on some species recently sequenced. Most importantly, we have conducted systematic comparative analysis to reveal their basic characteristics, conserved motif, duplications and losses, evolutionary, expression patterns, and phylogenetic relationships.

Our analysis showed that more *Hsf* family genes were detected in most higher plants than in lower plants. This might be due to the multiple WGD or WGT events in plants, especially higher plants, leading to the production of more heat-resistant genes to adapt to high temperatures [46–48]. Interestingly, the three species with the highest percentage of *Hsf* family genes were all Brassicaceae species. This phenomenon might also relate to the WGD and WGT events of the Brassicaceae species [49–51]. The number of *Hsf* gene losses was more than gene duplication in most Lycopodiophyta, Gymnospermae, and Angiospermae species, which indicated that the *Hsf* family genes were lost after the WGD or WGT events in higher plants.

*A. thaliana* is the typical model plant that has provided the reference for studying gene function and evolution in other plants [52]. In this study, we constructed the interaction network for *Hsf* family genes and their target genes in *Arabidopsis*. Then, enrichment analysis of target genes indicated that the *Hsf* gene family interacted with other transcription factors, such as AP2, WRKY, and Myb_DNA-binding, in response to heat stress. Furthermore, the global expression pattern of the *Hsf* gene family in *Arabidopsis* was explored. All of these results in the model species *Arabidopsis* will provide a guide for studying the *Hsf* gene family in other species.

*B. rapa* is one of the most economically important *Brassica* species and is the main vegetable crop worldwide [53, 54]. *B. rapa* (Chinese cabbage), is a typical horticultural plant. It experienced a WGT event after it diverged from *Arabidopsis* [50, 55]. Here, we conducted *Hsf* gene family analysis using the pan-genome of 18 *B. rapa* accessions reported recently [53]. The syntenic analysis showed that 0–3 copies of *Arabidopsis Hsf* family genes were detected in the three subgenomes of 18 *B. rapa* accessions. The results lay the foundation for better research on the function of duplicated genes.

Furthermore, we performed expression pattern analysis of *Hsf* family genes and several *Hsp* gene families using expression datasets from different tissues and under different heat treatments in *B. rapa*. Then, the interaction network between *Hsf* gene family and *Hsp* gene families was constructed in *B. rapa*, and several core genes were detected in the network. This network well reveals the regulatory relationship of *Hsf* and various *Hsp* gene families, and also provides a guide for experimental studies on the functional interaction of these family genes in future.

Finally, we constructed a database (Hsfdb) for all identified *Hsf* family genes in 111 representative plants. Compared with the PlantTFDB, we have also performed *Hsf* gene family analysis on some species recently sequenced.

Second/next-generation sequencing (NGS) has tremendously improved sequencing output, and has made genome sequencing much faster and cheaper than Sanger sequencing. However, NGS technology has several drawbacks, most obviously the short reads [56]. Third-generation long-read sequencing overcomes the limitation of short-read sequencing, and can produce high-quality genome assemblies. Therefore, third-generation sequencing has the ability to resolve repeat sequences and large chromosomal rearrangements [56, 57]. Second-generation sequencing leads to incomplete genomes of species, and may lead to the loss of some genes. However, the most influential region should be the repetitive sequence, and the repetitive region contains a relatively small number of genes. Moreover, we use the latest version or the version with higher assembly quality when the species has multiple versions of the genome. Of course, with the development of three-generation sequencing and the reduction of costs, we believe that nearly complete genomes will be obtained for more and more species, just like the human genome and the *Arabidopsis* genome recently reported [58, 59]. Our database will be continuously updated with updates of the species genome.

In conclusion, we performed comprehensive analyses of the *Hsf* gene family in 111 horticultural and other representative plants, especially for the study of the horticultural plant *B. rapa*. We also constructed a database for all identified *Hsf* family genes. This study will serve as a useful resource for future studies on the biological function and evolutionary history of the *Hsf* gene family.

## Materials and methods

### Sequence collection

The protein sequences of examined plants in Fasta format were downloaded from the Ensembl database (http://useast.ensembl.org/index.html). The 18 *B. rapa* pan-genome sequences were downloaded from the BRAD database (http://brassicadb.cn) [60]. Alternative splice sequences were deleted using a custom Perl script to ensure the non-redundancy of the sequences that were used. The phylogenetic trees of species were made using the iTOL website according to the relationship of species in NCBI taxonomy [61].

### *Hsf* gene family identification and statistics

The *Hsf* family genes were identified using the Pfam database (PF00447) with an e-value <1e^−4^ [62]. Furthermore, the SMART and CDD databases were used to conduct domain validation to ensure the results’ accuracy [63, 64]. The SMART and MARCOIL databases were also used for DBD and HR-A/B domain detection, respectively [63, 65]. Violin plots with a boxplot of the *Hsf* family gene number of each kingdom were drawn using the ggviolin function in the ggpubr package of the R program (https://cran.r-project.org/web/packages/ggpubr/index.html).

### Phylogenetic relationship construction

Firstly, the protein sequences of the *Hsf* gene family were aligned by Mafft v7.471 software with maxiterate at 1000 [66]. The maximum likelihood tree was constructed using FastTree (v2.1.11) software [67]. The JTT (Jones–Taylor–Thornton) model was used, and bootstrap replications were set at 1000.

### Conserved motif identification and gene duplication and loss inference

The amino acid sequences of the *Hsf* family genes from seven representative plants were used for motif analysis using Multiple Expectation Maximization for Motif Elicitation (MEME) with the default parameters [68]. *Hsf* family gene duplication and loss were identified by Notung2.9 software [69]. All of this information on the phylogenetic trees was illustrated by the iTOL program [61].

### Duplication type detection for *Hsf* family genes

The collinearity of *Hsf* family genes among seven representative plants was detected using MCScanX software [70]. Firstly, the protein sequences of these species were aligned using the Blastp program with an e-value of 1 × 10^−5^. Then, the collinear blocks were detected using MCScanX with the default parameters. Finally, the gene duplication types were identified using a duplicate_gene_classifier program from MCScanX software. Significance analysis of the duplication type for *Hsf* family genes compared with whole-genome genes was conducted using the χ^2^ test (*P* < .01).

### Target gene identification and interaction network construction

The target genes of the Hsf gene family in *Arabidopsis* were identified using the integrated gene regulatory network (iGRN) database (http://bioinformatics.psb.ugent.be/webtools/iGRN/) with score ≥0.60 [71]. In this study, we divided the target genes into downstream and upstream genes. The downstream genes were regulated by *Hsf* family genes, while the upstream genes regulated the *Hsf* family genes. The interaction network between *Hsf* family genes and target genes was constructed using Gephi software (v0.9.2) with a continuous graph layout algorithm ForceAtlas2 (https://gephi.org) [72].

### Functional annotation and enrichment analysis of target genes

Functional annotation of the above-identified target genes and all genes of *Arabidopsis* was performed using the Pfam database (http://xfam.org) [73]. Then, enrichment analysis was conducted by comparing four groups: target genes with a related functional term; target genes with annotation; all genes with a related functional term; and all genes with annotation in *Arabidopsis*. Finally, the scipy package of Python was used to perform enrichment analysis [74]. The *P*-values obtained by significance analysis were further corrected using the Bonferroni method in the R program. The corrected *P*-value (*q*-value) <.05 and fold change >2 were used to define significant enrichment terms. The TBtools program was used to generate the Venn diagram, which indicated the specific or shared enriched terms for the downstream and upstream target genes [75].

### Gene expression data retrieval and analysis

The large-scale expression datasets under various stresses and developmental stages were collected from the website of the *Arabidopsis* eFP browser (http://www.bar.utoronto.ca) [76]. We collected 154 samples from 18 groups under various abiotic stresses, 70 samples from 27 groups under various biotic stresses, and 47 samples from different developmental stages of *Arabidopsis*. Then, we explored the expression of the *Hsf* gene family using these large-scale biological datasets. The TBtools program was used to generate a heat map according to cluster analysis of the expression values [75].

For *B. rapa*, the expression dataset under heat stress was obtained from the Genome Sequence Archive (GSA) in the BIG Data Center (accession number CRA002707) according to our previous report [54]. Heat treatment was conducted at 38°C for 1 hour (T1), 4 hours (T4), 8 hours (T8), and 12 hours (T12); the control was without heat treatment. The expression dataset in different tissues was obtained from Gene Expression Omnibus (GEO) in NCBI (accession number GSE43245) according to a previous report [77]. The expression values were normalized as fragments per kilobase of transcript per million mapped reads (FPKM) [78]. Integration of the phylogenetic tree and creation of the expression heat map were performed using the iTOL program [61].

### *Hsp* gene family identification and interaction network construction

The six main *Hsp* family genes were identified in this study. Among these, *Hsp20*, *Hsp70*, and *Hsp90* family genes were directly extracted from the Pfam database using the identifier numbers PF00011, PF00012, and PF00183, respectively (e-value <1e−4) [62]. The *Hsp40*, *Hsp60*, and *Hsp100* family genes were identified using the hmmsearch program because there was no Pfam identifier number [79]. PCCs between *Hsf* and *Hsp* were calculated using in-house Perl scripts according to the gene expression value under heat treatment. The positive and negative regulatory relationships were defined as PCC > .95 and PCC < −0.95, respectively [80, 81]. The interaction network between *Hsf* and *Hsp* was constructed using Gephi software [72].

### Identification and visualization of *cis*-acting elements

The promoter sequences were extracted from the 2 kb upstream of the translation initiation site of each *Hsp* family gene. Then, the *cis*-acting elements were estimated in these promoter sequences using PlantCARE [82]. The visualization of *cis*-acting elements in each *Hsp* promoter region was realized by TBtools software [75]. HSEs were detected from the promoter sequence of each *Hsp* family gene using the in-house Perl script. The consensus sequence of GAAnnTTnnnGAA was used to predict HSEs according to previous reports [83–85].

### Pan-genome analysis of *Hsf* gene family in *B. rapa*

We performed *Hsf* gene family identification and analysis using the pan-genome of 18 *B. rapa* accessions reported recently [53]. The *B. rapa* genome was further divided into three subgenomes, comprising the less fractioned subgenome (LF) and the more fractioned subgenomes (MF1 and MF2), according to a previous report [86]. Thus, we explored the absence or presence of *Hsf* family genes in three subgenomes of *B. rapa*.

### Database construction

The *Hsf* database was built using several software packages, including Microsoft .NET Framework 4.5, MySQL database management, PHP, HTML, CSS, and JavaScript. The collected data were processed using a Perl program for conducting biological data analysis. The interactive web interface was constructed to enable users to conveniently access our database. HTML, PHP, and JavaScript were used to transmit query requirements and extract *Hsf* gene family-related data from the MySQL database to show in the report pages. The interactive plotting system was made using d3.js and the nvd3 helper library [87].

## Supplementary Material

Web_Material_uhac035

## Data Availability

All materials and related datasets in this study are available in our *Hsf* database (http://hsfdb.bio2db.com).
