# Supplementary material for: Large-scale analyses of heat shock transcription factors and database construction based on whole-genome genes in horticultural and representative plants
Source: Hortic Res. 2022 Feb 19;9:uhac035. doi: 10.1093/hr/uhac035 (PMC9123238; doi:10.1093/hr/uhac035)
Supplement: Web_Material_uhac035 [file web_material_uhac035.zip › Supplementary Figures 1-4.pdf]

Supplementary Figures 1-4

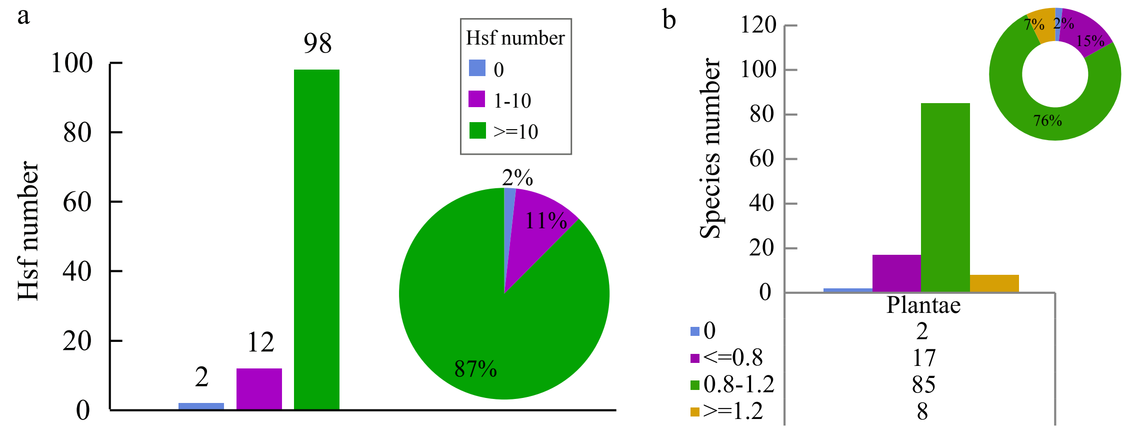

Fig. S1 (a) The statistics of *Hsf* family gene number and percentage for three categories (*Hsf* gene number: 0, 1-10, >=10) in plants. (b) The statistics of *Hsf* family gene length ratio (Average length of *Hsf* genes/Average length of all genes), and their percentage for four categories (length ratio: 0, <=0.8, 0.8-1.0, >=1.2) in plants.

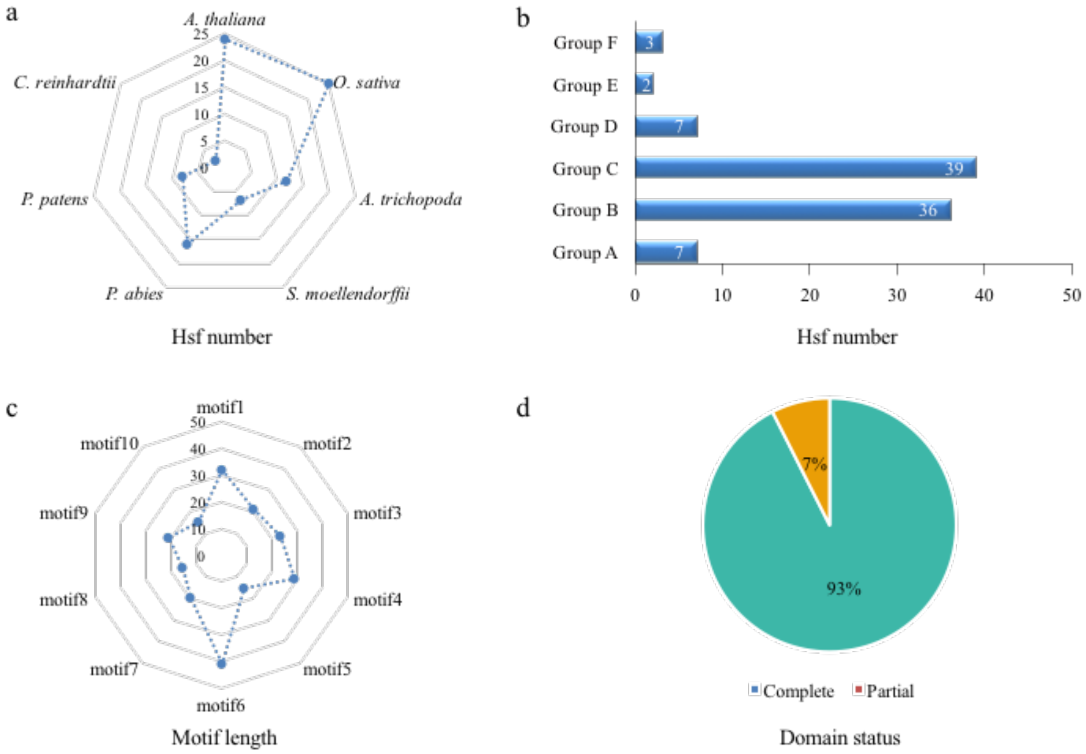

Fig. S2 The number, classification, conserved motif, and domain analysis of *Hsf* family genes from 7 representative species. (a)The number of *Hsf* family genes in each species. (b) The number of *Hsf* family genes in each group. (c)The length of each motif. (d) The percentage of complete and partial domain.

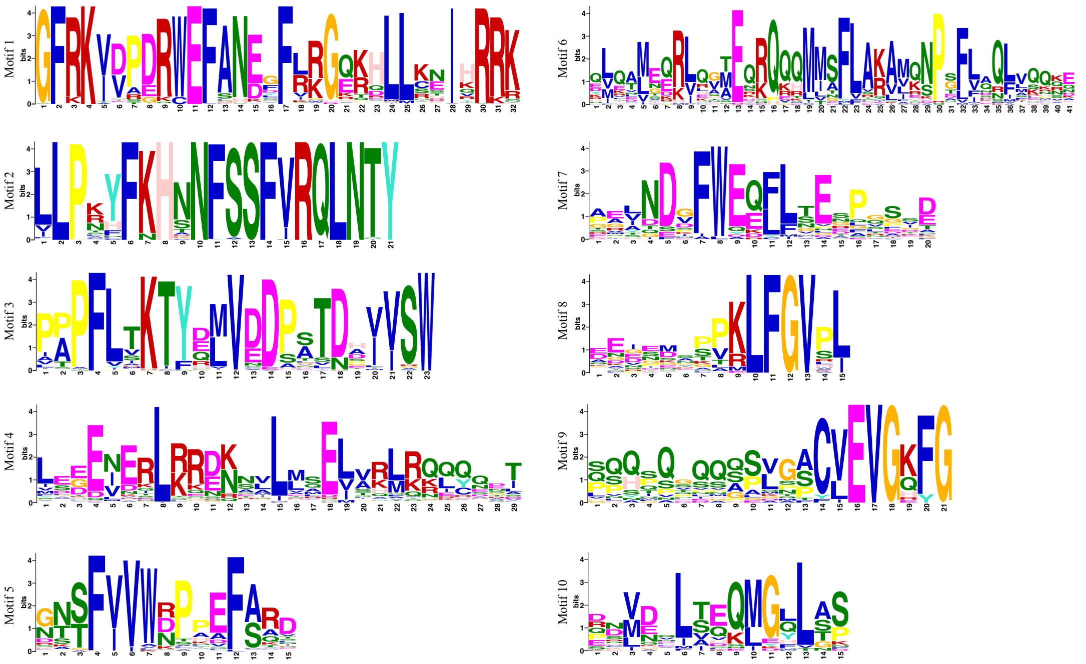

Fig. S3 The conserved sequences of each motif for *Hsf* family genes from 7 representative species.

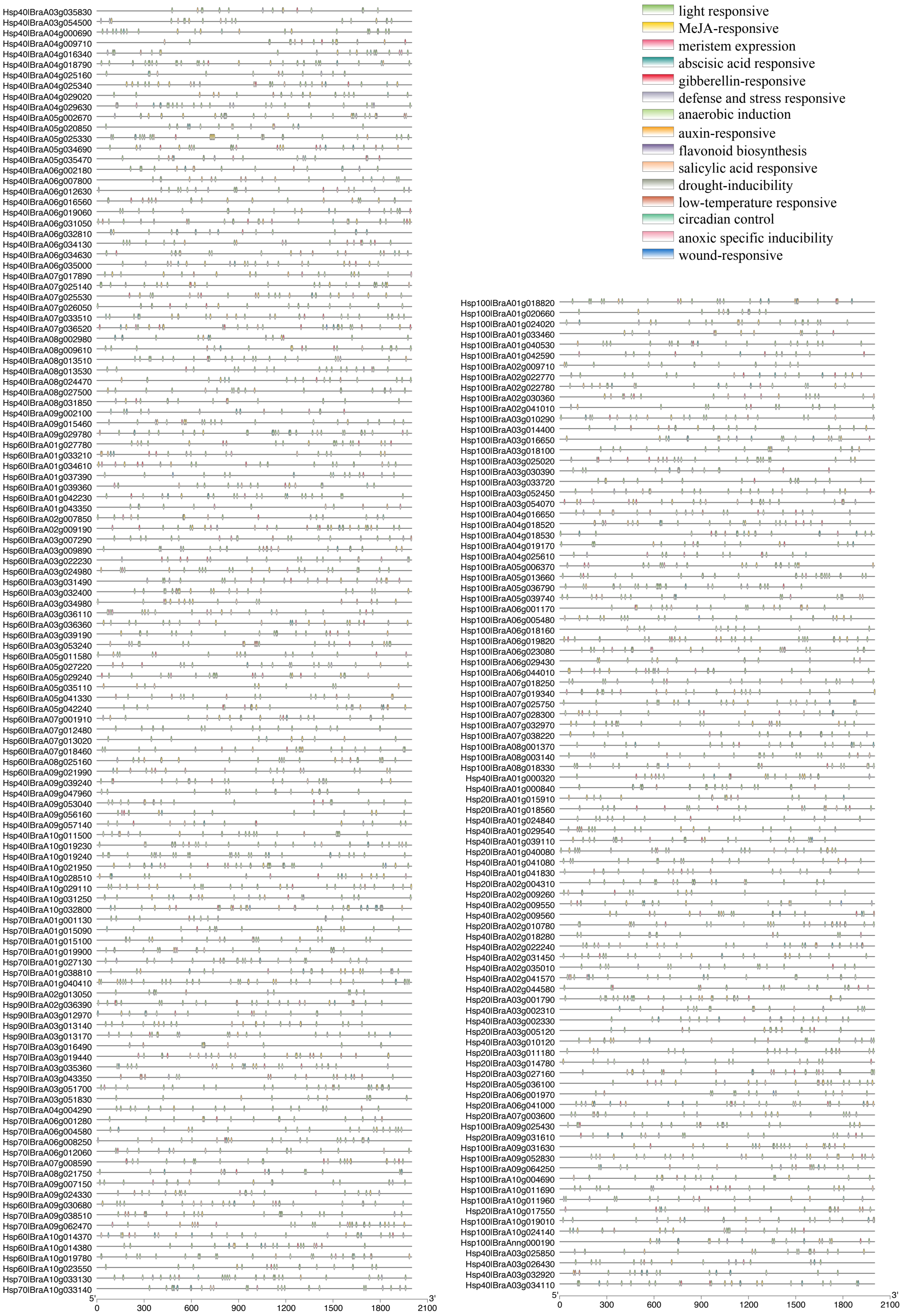

Fig. S4 The cis-acting elements in the promoter of each *Hsp* family gene in the network.
